# Representation models and processing operators for quantum informational multi-media

**DOI:** 10.1371/journal.pone.0313294

**Published:** 2025-01-16

**Authors:** Yajun Li

**Affiliations:** College of Information Science and Technology & College of Artificial Intelligence, Nanjing Forestry University, Nanjing, China; Stockholms Universitet, SWEDEN

## Abstract

To enhance the efficacy of multimedia quantum processing and diminish processing overhead, an advanced multimedia quantum representation model and quantum video display framework are devised. A range of framework processing operators are also developed, including an image color compensation operator, a bit plane inversion operator, and a frame displacement operator. In addition, to address image security issues, two quantum image operations have been proposed: color transformation operation and pixel blending operation. The research results indicated that the grayscale cost of the framework designed in this study was 33.8, the color cost was 40.5, and the total cost was 574 δ. In terms of color, the distribution of image elements in the red, green, and blue (RGB) channels was more balanced. In summary, the quantum video display framework has significant advantages in processing efficiency and image security. Compared to previous studies, the model proposed in this study exhibits higher processing speed and better processing quality in the face of complex image and video data. It effectively addresses the limitations of existing processing techniques while addressing emerging image security issues.

## 1. Introduction

Quantum information science is an interdisciplinary field that draws upon the principles of quantum physics, information science, and mathematics to gain a deeper understanding of the fundamental processes of information processing and to develop innovative solutions for future generations of information technology. Just as classical computers need classical information representation models (RM) and processing operators (PO), quantum information processing needs its own RM and PO. After many areas of research such as quantum communication and quantum computation, research in related fields is gradually shifting to quantum informatics multi-media (QIM) direction [1–3]. In recent years, with the rapid development of multi-media technology and network technology, QIM technology has received widespread attention and research from many scholars around the world, such as in the field of quantum imaging, focusing on quantum image (QI) processing, image processing, etc. [4]. The efficient information processing performance, flexible information representation, and powerful information protection capabilities of quantum technology provide new solutions for multi-media information processing [5,6]. However, there is currently limited research on QIM related theories, and it is necessary to combine research on QIM, RM, and PO [7–9]. Therefore, in order to optimize the processing efficiency of multi-media information and reduce processing overhead, an efficient and low-power quantum information multi-media processing framework has been designed and developed. The utilization of quantum mechanisms for the encoding of video frames has the potential to enhance the efficiency of multimedia data processing. Overall, these measures aim to explore new ways for quantum information science to solve complex multi-media information processing problems, providing new theoretical foundations and technical means for future research and applications. The first part of the study introduces the research purpose of combining quantum technology and multi-media. The second part designs the multi-media quantum RM. The third part examines the effect of the model. The fourth part gets the conclusion of the study.

The contributions of this study is: (1) An efficient and low-power quantum information multi-media processing framework is proposed. This framework can optimize the processing efficiency of multi-media information and reduce processing overhead. This new processing framework provides a new solution for multi-media information processing problems. (2) Research has explored new ways for quantum information science to solve complex multi-media information processing problems. The Results provide new theoretical foundations and technical means for future research and applications, and offer new directions for the development of quantum information science in the field of multi-media processing.

Currently, there are few established frameworks for quantum video representation, and the corresponding POs are still underdeveloped. The majority of models and algorithms for QI and video representation remain theoretical, having yet to be implemented in actual quantum computers or real quantum simulators. These simulators are designed to emulate the parallel computing rules of quantum computers for the purpose of conducting simulations in the real world. Therefore, this study proposes an efficient quantum video representation framework and conducts display simulation experiments to verify its performance, which is a beneficial supplement to related fields.

The contribution of the research and design model is that it can be used for real-time processing and analysis of surveillance video, improving the accuracy and speed of intelligent surveillance image recognition and motion detection. In the context of data security in military and government departments, research and design models can serve to protect sensitive information and prevent data tampering by balancing the distribution of RGB three-channel image elements, thereby enhancing the security of image data. In addition, in film and animation production, research and design models can improve rendering and post-processing efficiency, reduce production time and cost, and ensure high-quality visual effects.

The model presented in this article is primarily designed to address the challenges of low efficiency and high cost in multimedia quantum processing through the implementation of a quantum multimedia representation framework. However, due to the fact that the model is a universal model, it has not yet been modularized to meet specific video processing requirements. In the future, consideration will be given to further solving the low demand for specialized video processing through modular design of universal models.

The study is divided into four parts. The first part introduces the research objective of solving the cost and efficiency problems of video processing models. The second part designs a multi-media quantum RM. The third part analyzes the effects of multi-media quantum RMs under different situations. The fourth part draws research conclusions.

## 2. Related works

Quantum imaging is one of the focuses of research in the field of quantum information, focusing on the processing and analysis of QI information. Wang Z et al delved into the latest research in QI processing from the basic architecture and mathematical theory of quantum computing. The course covered a range of topics related to the presentation of QIs, the various methods for modifying their morphology, the utilization of image links, the delineation of image boundaries, the segmentation of images, the cleansing of images, and the compression of images, among other subjects. Their firm view was that despite the fact that QI processing still had problems that had not been fully solved, its advantages and potential results for future research were still worth looking forward to [10]. Wang X et al. provided a refreshed approach to QI encryption that was rooted in super chaotic systems and quantum revolving doors. By using a serialization operation using the quadruple matrix of the generated chaotic system, the study employed a unique method to produce a semi-ciphertext image by obfuscating the key and the plaintext. Their results showed that the security of this method was astounding [11]. Focusing on optimizing the quality of computed tomography images, Sartoretti T et al. investigated reconstruction algorithms for the reconstruction of computed tomography (CT) images diagnosed by clinical photon counting detectors. As a result of their research, they identified a very strong reconstruction algorithm that reduced noise and improved image brightness and lesion visibility without destroying the image texture or CT attenuation values, thus improving the image quality [12]. Hybrid machine learning approach was also used for medical image analysis by Sohail A team who found a new algorithm which surpassed the existing algorithms at the theoretical level and was able to categorize medical images with higher efficiency. The study set rigorous and efficient steps for cleaning, segmentation, statistical analysis and classification of medical images [13]. Shen H et al. developed a novel fluorescent dye with a remarkable 31.5% fluorescence quantum yield. The most significant contribution of this research is the invention of a novel interaction method that markedly enhances the stiffness of the model in the energy storage state, thereby achieving an exceptionally high fluorescence quantum output. Therefore, the emergence of a new type of biological imaging technology using this fluorescent dye provides new ideas for the design of materials and biological imaging technology in the future [14].

The research on quantum information models and related quantum systems is currently the focus of quantum information research and the key to QIM research. Dong D et al. investigated the principles of evaluation and control of quantum systems, which were critical for practical applications. The study detailed the difficulties faced in quantum evaluation, control, and learning about these new fields, and demonstrated the potential opportunities. The areas they investigated covered quantum state evaluation, quantum parameter identification, quantum filtering, quantum open-loop control, quantum inverse control, and also machine learning of quantum systems. In addition, the study discussed the field of quantum AI [15]. Lee et al. designed a novel automation system based on quantum programming, which can automatically master operable functions and write concise code for most scenarios. In addition, the system is able to quickly learn and adapt when introducing new operations, with a greater emphasis on code simplicity. Experimental results show that compared to existing systems, the new system can generate programs with 40 times fewer operations [16]. Mihalcea L C et al. focused on Demazure-Lusztig operations on biased labeled manifolds. The study confirmed that these operations generate the Chern-Schwartz-MacPherson classes of Schubert cells or their motivic Chern classes for all locally labeled manifolds. Their study provided valuable insights into the function of the concomitant differential operations in homology and K-theory and their actions in endomorphism classes [17]. Fields C et al. investigated a class of quantum systems with sufficient degrees of freedom within the framework of free energy theory. It concluded that these systems were able to reach self-validation by minimizing Bayesian prediction errors. The study indicated that quantum FEP was essentially analogous to the unitary metric principle and postulated that biological systems may utilize quantum coherence as a computational and communication resource. Additionally, the study proposed several challenging topics for future research [18].

At present, research shows that quantum technology, chaotic encryption, and intelligent healthcare are developing rapidly in their respective fields. Research on chaotic encryption tends to adopt fractional-order chaotic systems, which improve the security and efficiency of encryption algorithms by increasing control parameters and complex dynamic properties [19–22]. In the field of intelligent healthcare, the quantum Internet of Things has also been deeply established, which meets the requirements of intelligent healthcare for data security, processing, and diagnosis through technologies such as quantum computing and quantum communication, and improves the reliability of personalized diagnosis [23,24]. In addition, quantum secure computing and quantum blockchain technology can improve the efficiency and security of data aggregation and communication, effectively reducing computing and communication costs [25]. In contrast, this study focuses on quantum multimedia processing, in particular the design of representation models and processing operators for quantum video. This area is relatively rare in existing research, but its application prospects are wide. The advantage of this study lies in achieving low cost and high efficiency in multimedia processing, focusing on multimedia processing efficiency and cost control, and applicable to various fields such as medical image processing, intelligent surveillance, and military image encryption, with broad prospects for promotion.

In recent years, the research trend in the field of QIs has diversified, and various methods and techniques have emerged, prompting the field to develop in a broader direction. Research related to multi-media mainly focuses on QI processing, multi-media RM, and PO. Various teams are targeting different problems and using different methods to conduct research. Some studies focus on theory construction, others on practical application, and still others on self-optimization of the system. This demonstrates the breadth and depth of current research. In the realm of QI processing, researchers aim to improve both the display and processing methods, as well as the security encryption technology of QI. This will provide a more secure means of information transmission and encryption for QIM. Quantum technology is increasingly utilized by researchers for image analysis and processing to enhance image quality. This is especially noticeable in the field of medical image analysis and processing, where researchers are developing more precise and effective methods for diagnosing lesions and interpreting medical images. Therefore, this research focuses on the combination of quantum processing and multi-media. This combination is expected to provide a new theoretical basis and technical means for automating new media technology and quantum systems in the future, promoting further development in this field.

## 3. Design of high-dimensional quantum multi-media content characterization models

The study focuses on multi-media quantum RM design, including multi-media quantum representation framework, multi-media editing operator design, and multi-media color operator design. First, the study designed advanced multi-media quantum representation model (AMQRM). Then, the video PO design is explored, including image color complement operator (ICCO), frame shift operator and bit plane inversion operator. They are used to realize the color tone mega-variation, adjust the playback order, and simple encryption of video, respectively. Finally, the study also designs two QI processing operations-color transformation operation and pixel mixing operation-constructed based on an efficient quantum video representation architecture model.

### 3.1. Design of a multi-media representation framework for quantum coding mechanisms

The idea of quantum video production is an easy to understand but technically complex process. The basic concept derives from the foundations of cinematography and videography—a series of static images or "frames" are played continuously at a specific rate to create a dynamic effect by means of the transient effect of the human eye’s vision [26–28]. Similarly, the basic construction of a quantum video is a sequence of frames, but instead of ordinary images, these frames are quantum states encoded with the help of quantum mechanical principles. The design conception of this production framework originated from previous research and consists of two main parts, a model description of each frame and how to project these photos in a sequential and time-stamped manner [29,30]. The model describing each frame of a photo is embodied by quantum bit parallel processing, which eliminates the data bottleneck in traditional multi-media processing. Sequential playback of photos with time stamps is then realized by superposition and entanglement of quantum states, which further improves the speed and efficiency of information processing. The AMQRM quantum video presentation framework designed in this study is an advanced method for describing 24 frames of video in one second. Different from the traditional video representation, this model is presented in the form of quantum bitmap, which can process a large amount of image data at one time, greatly improving the processing efficiency. With the unique properties of quantum mechanics, including superposition and entanglement, AMQRM not only allows for faster processing of video data, but also increases the security and immunity of the system. The AMQRM quantum video presentation frames depend on the bit-plane representation quantum image model (BRQI). The abbreviation table during the research process is shown in Table 1.

**Table 1:** Nomenclature.

Abbreviation	Full name
BRQI	Bit-Plane Representation Quantum Image Model
CT	Computed Tomography
FFQM	Full Frequency Quantum Model
MFQM	Multi-Frequency Quantum Model
QVNEQR	Quantum Video Non-Equilibrium Quantum Representation
QVNCQI	Quantum Video Non-Equilibrium Color Quantum Information
AMQRM	Adaptive Multi-Quantum Representation Model
RM	Representation models
PO	Processing operators
QIM	Quantum informatics multi-media
QI	Quantum image
CT	Computed tomography
IFDO	image frame displacement operator
IBIO	image bit-plane inversion operator
ICCO	image color complement operator
TTO	tone transformation operator
ICSO	image color mixing operator

As show in Table 1, this model decomposes a full frame of color photos into 24 black and white photos when presenting the image. This enables the presentation of the coordinate data of each pixel point, as well as the data of the bit plane sequence and the black and white data of the pixel corresponding to the bit plane sequence of each frame within the frame. The video representation in the frame is shown in Eq (1) [31].


$$|V_{t}(m)\rangle =\frac{1}{2^{m/2}}\sum_{t=0}^{2^{m}-1}|F_{t}(m)\rangle ⊗|t\rangle$$ (1)

In Eq (1), |*F*_*t*_(*m*)〉 denotes the frame image, |*t*〉 is the timing expression, and *m* denotes the number of quantum bits. |*F*_*t*_(*m*)〉 is calculated as in Eq (2).


$$|F_{t}(m)\rangle =\frac{1}{2^{n+5/2}}\sum_{B=0}^{2^{5}-1}\sum_{Y=0}^{2^{n}-1}\sum_{X=0}^{2^{n}-1}|B\rangle |G_{t(b,y,x)}\rangle ⊗|YX\rangle$$ (2)

In Eq (2), |*YX*〉 denotes the pixel coordinates, |*B*〉 denotes the plane sequence, and |*G*_*t*(*b*,*y*,*x*)_〉 denotes the corresponding plane sequence binary data. |*B*〉|*G*_*t*(*b*,*y*,*x*)_〉 is calculated as in Eq (3)[32].


$$\sum_{B=0}^{2^{5}}|B\rangle |G_{t(b,y,x)}\rangle =|B_{0})\rangle |G_{t(b_{0},y,x)}\rangle +\cdots +|B_{31}\rangle |G_{t(b_{31},y,x)}\rangle$$ (3)

**Fig 1:**
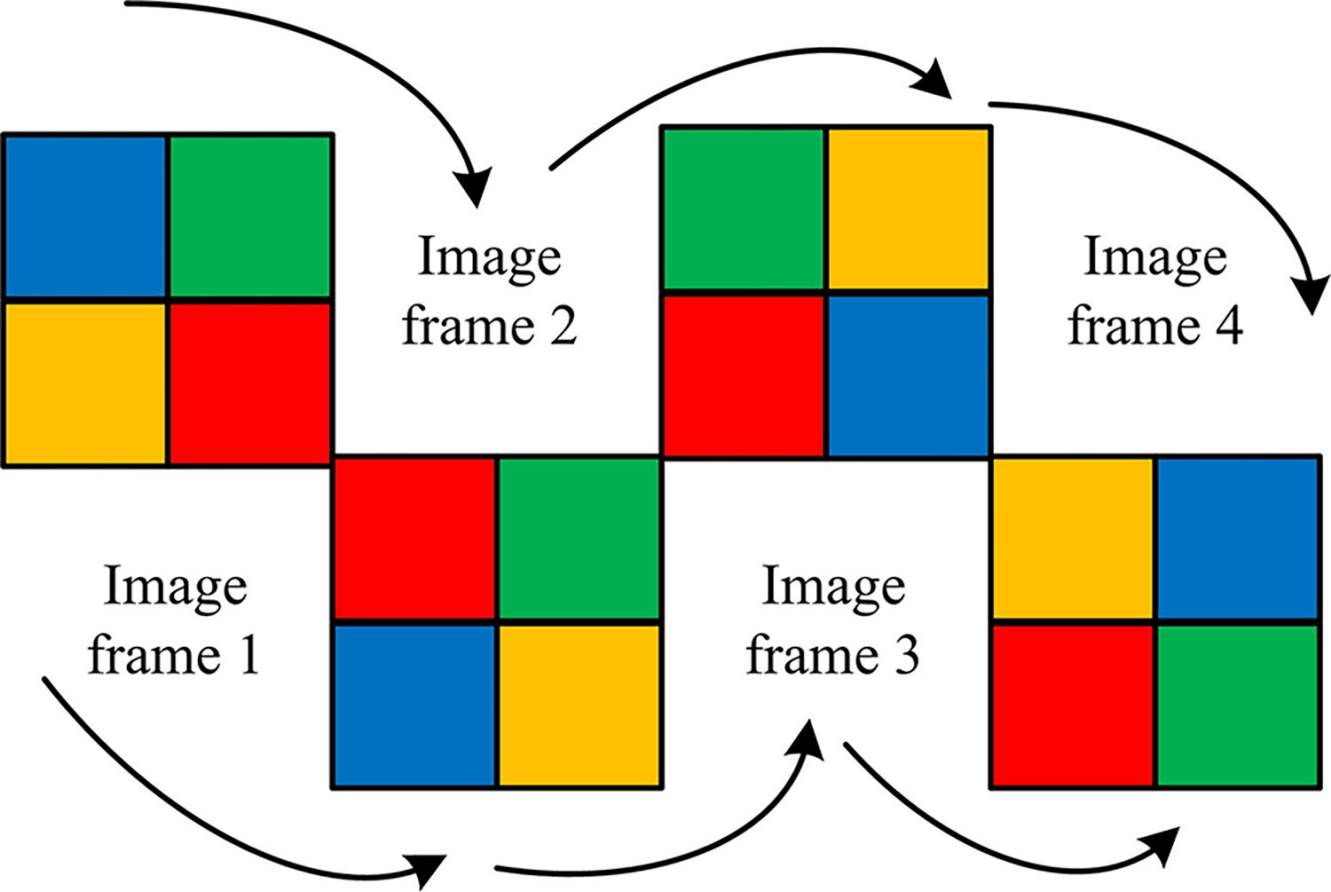
Four frame video example.

Both $|B_{0}\rangle |G_{t(b_{0},y,x)}\rangle$ and $|B_{31}\rangle |G_{t(b_{31},y,x)}\rangle$ in Eq (3) are 0. To gain insight into the presentation and qualities of the AMQRM framework, the study is elaborated using a minimized example video containing 4 frames. In this example, each frame is a 2×2 sized color image with two quantum bits representing the position information of each color image. Five quantum bits represent the bit plane sequence information, one quantum bit represents the pixel binary information under the bit plane sequence, and two additional quantum bits represent the four frames of the image. Therefore, 10 quantum bits are required to create an AMQRM video containing four frames with 2×2 size as shown in Fig 1.

In Fig 1, the initialization process of the AMQRM quantum video presentation framework can be divided into two steps. The first step is to initialize a quantum register of 2*n*+*m*+6 quantum bits to prepare the quantum initial state. The initial state is shown in Eq (4).


$${|\delta \rangle }_{0}={|0\rangle }^{⊗2n+m+6}$$ (4)

The initial quantum state is transformed into an intermediate quantum state by means of a quantum operator. The underlying quantum gate of the process is shown in Eq (5).


$$I=[\begin{array}{cc} 1 & 0\\ 0 & 1 \end{array}],H=\frac{1}{\sqrt{2}}[\begin{array}{cc} 1 & 1\\ 1 & ‐1 \end{array}]$$ (5)

The intermediate quantum state is as in Eq (6) [33].


$$|V_{1}\rangle (\left|{\delta \rangle }_{0}\right)=I⊗H^{2n+m+5}(\left|{0\rangle }^{2n+m+6}\right)$$ (6)

**Fig 2:**
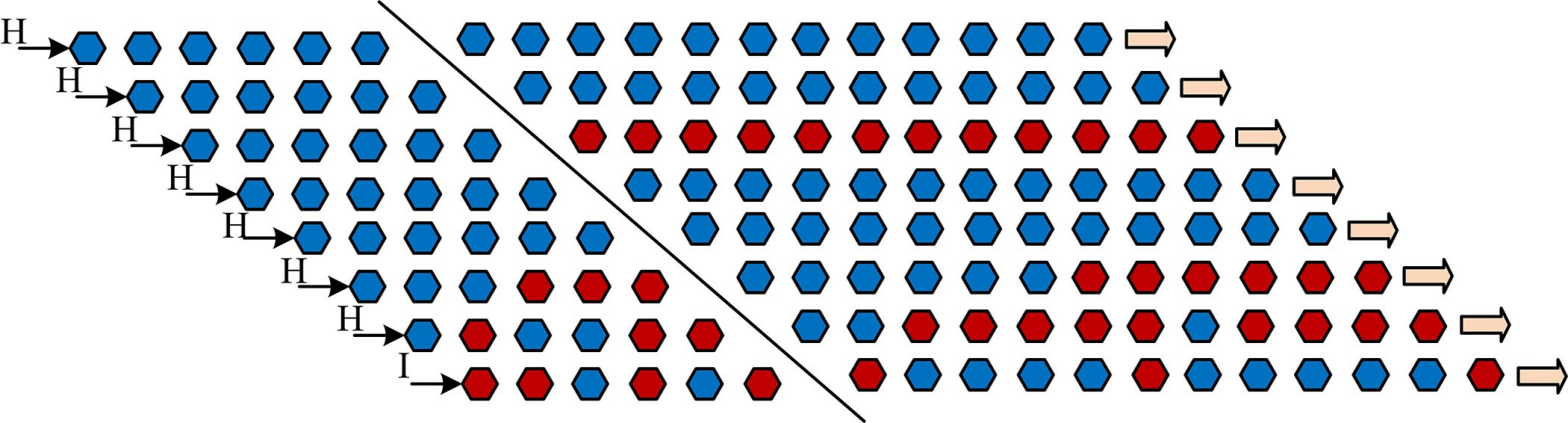
Video preparation intermediate state.

In Eq (6), *I*⊗*H*^2*n*+*m*+5^ denotes the quantum operator. At this juncture, the temporal data associated with the video, the pixel coordinates within the corresponding frame, and the bit plane information have been encoded into the intermediate quantum state. However, the color information remains at a value of zero. The quantum intermediate state is shown in Fig 2.

Fig 2 shows the preparation process of a 4 frame 2×2 size video sample, which expresses the pixel information of the original video. The color information is sequentially written into the intermediate state using a sub operator composed of the *CONT* gate and other basic gates. In the original video, each frame is a color image of size 2n × 2n. Therefore, it takes 2^*n*+5^ sub-operators to write the information of one frame into the intermediate state. Since the whole video has a total number of frames, 2^*n*+*m*+5^ sub-operators are required for the production of the whole video. The sub-operators are as in Eq (7).


$$V_{TBYX}=(I⊗\sum_{k=0}^{2^{m}-1}\sum_{i=0,j=0,ji\neq YX}^{2^{2n}-1}\sum_{b=0}^{2^{5}-1}|kbji\rangle \langle kbji\rangle )+\Omega_{TBYX}⊗|TBYX\rangle \langle TBYX|$$ (7)

In Eq (7), Ω_*TBYX*_ is the quantum assignment of the corresponding pixel on the corresponding bit plane sequence operator. *YX* denotes the operator corresponding to the pixel that completes the quantum of the video, as in Eq (8) [34].


$$V_{2}=\prod_{t=0}^{2^{m}-1}\prod_{yx=0}^{2^{n}-1}\prod_{b=0}^{2^{5}-1}V_{TBYX}$$ (8)

**Fig 3:**
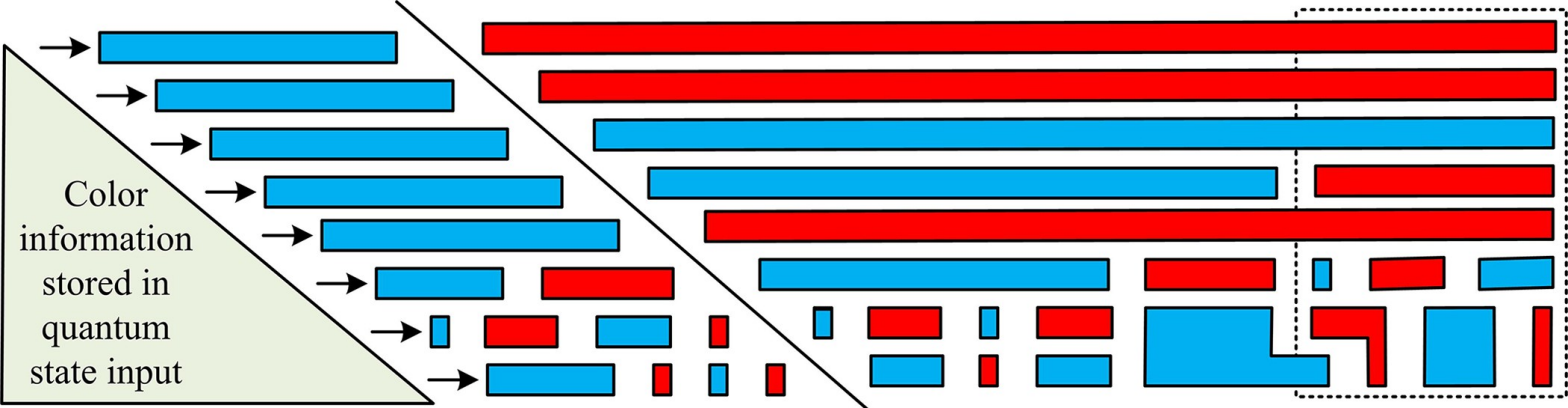
Quantum state storage steps.

The process of depositing the quantum state and completing the video is shown in Fig 3.

Fig 3 illustrates the process of storing quantum states and completing the video. The model sets the color information of the corresponding pixel’s position plane through an operator and flips the information through the *CONT* gate. As a result, the image in the model is considered a combination of binary images, and only one quantum bit is required to represent the color of the information.

In addition, the overhead of the quantum lines also needs to be taken care of during the production of quantum videos in the AMQRM framework. The actual time complexity will not exceed *O*((2*n*+*m*+5)2^2*n*+*m*+5^). This way the AMQRM model will be able to be able to produce quantum videos efficiently with limited overhead.

In the quantum state storage phase, it is essential to initialize the quantum register and verify that its quantum bit count is sufficient to accommodate the pixel and color data of each frame. Secondly, the quantum register must be initialized to an all-zero state, and the initial quantum state must be transformed into an intermediate quantum state by applying quantum operators. This process results in intermediate quantum states that contain time information, pixel position information, and bit plane information. It is necessary to use sub-operators to sequentially inscribe the chromatic data of interest into the intermediate quantum state. It is of paramount importance that the chromatic data of each individual frame is accurately recorded.

Once the position and color information of every pixel in the intermediate quantum state has been recorded, the quantum state can be used to represent every frame of the entire video. The system must ensure that all data is stored accurately. Once the quantum state storage process is complete, additional quantum operations are required to decode the quantum state into video frames, construct the final video output, and convert it into a format that can be recognized by the human eye or conventional devices.

### 3.2. Integration of quantum multidimensional video editing operations and operator optimization design

The research mainly focuses on the video PO architecture based on AMQRM quantum video performance. This design is of great importance in the field of video processing because the simplicity of the operation can have a wide range of impacts and convey deeper meanings to the viewers. The research will explore the three aspects of ICCO, image frame displacement operator (IFDO) and image bit-plane inversion operator (IBIO). First, the study explores the design of ICCO. In animation and video, the presentation of reminiscent or dangerous situations is often observed, in which the overall hue of the video tends to contrast greatly, or even diametrically, with the hue of the previous scene [35]. This effect can be achieved by the ICCO operator, which is an image color complementary operation via color quantum bit inversion to achieve a huge change in the hue of a quantum video. The ICCO operator is shown in Eq (9) [36].


$$U_{COC}=X⊗I^{⊗2n+m+5}$$ (9)

**Fig 4:**
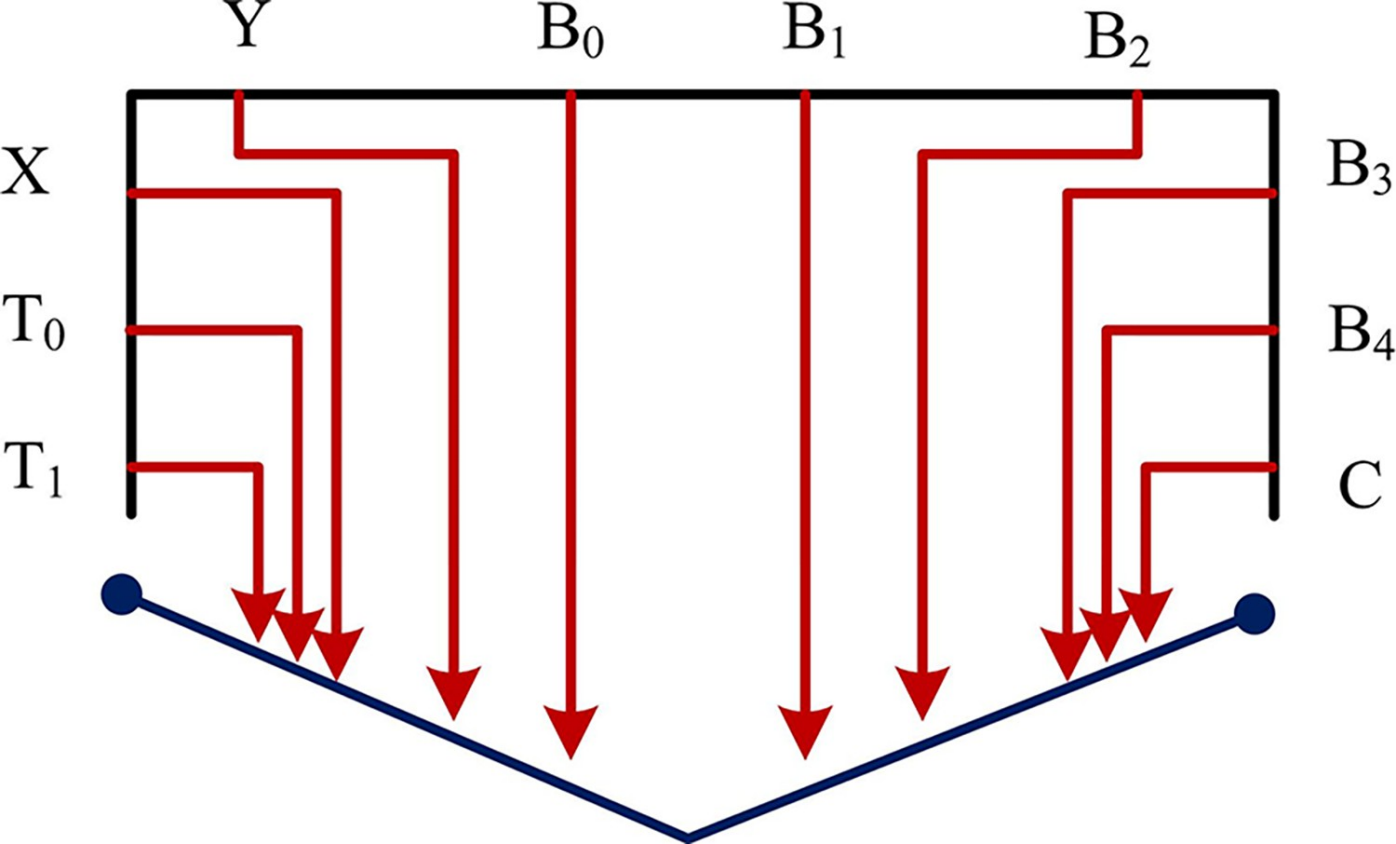
ICCO operator circuit.

The ICCO operator line is shown in Fig 4.

Fig 4 displays a simulated image of the circuit preparation, where the red lines indicate the operator-prepared circuit, and the letters represent the necessary quantum bits. In life, changes in the order of words can lead to different meanings. A comparable phenomenon is observed during the editing of video and animation. A modification of the playback sequence, specifically the order in which frames are presented, results in a change in the representation of the video [37,38]. Therefore, it is necessary to design an operator capable of adjusting the playback order of arbitrary frames. This is to be achieved by means of a frame displacement operator, which adjusts the playback order by changing the time index of two frames in the video, thus effecting a change in the representation of the original video. The frame displacement operator is shown in Eq (10) [39–41].


$$U_{IFDO}=Swap_{t_{i},t_{j}}⊗I^{⊗2n+5}$$ (10)

**Fig 5:**
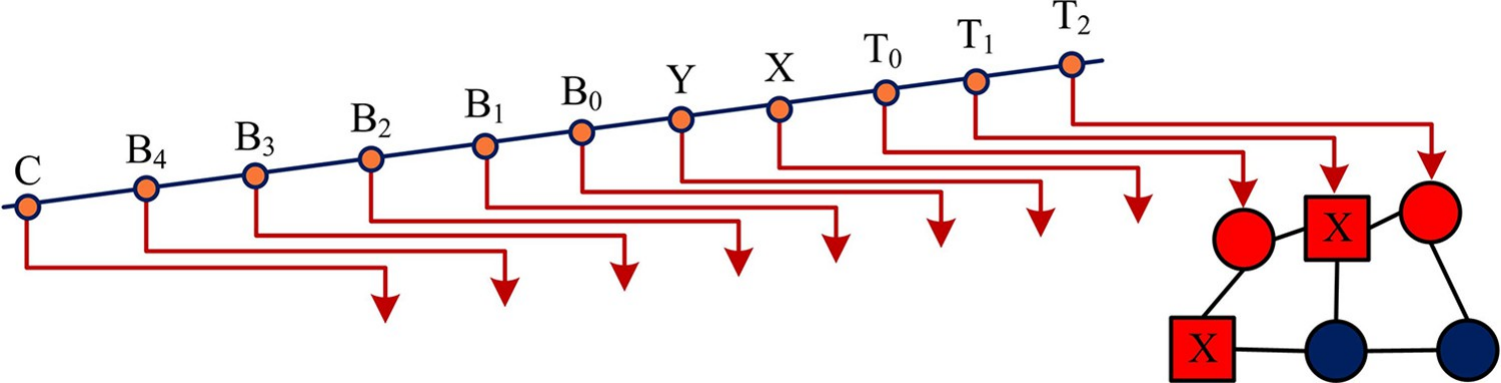
IFDO operator circuit.

In Eq (10), $Swap_{t_{i},t_{j}}$ is the frames the pair-wise commutator. The IFDO operator line is shown in Fig 5.

Fig 5 displays the circuit diagram for the IFDO operator, which is divided into three main parts. Toffli gates are used in each part to exchange the first and third frames, the fourth and sixth frames, and the seventh and eighth frames. In addition, encryption of the video is a key issue for copyright reasons. To realize the simple encryption of quantum video, the bit plane inversion operation operator can be used as shown in Eq (11).


$$U_{IBIO}=X^{⊗5}⊗I^{2n+m+1}$$ (11)

**Fig 6:**
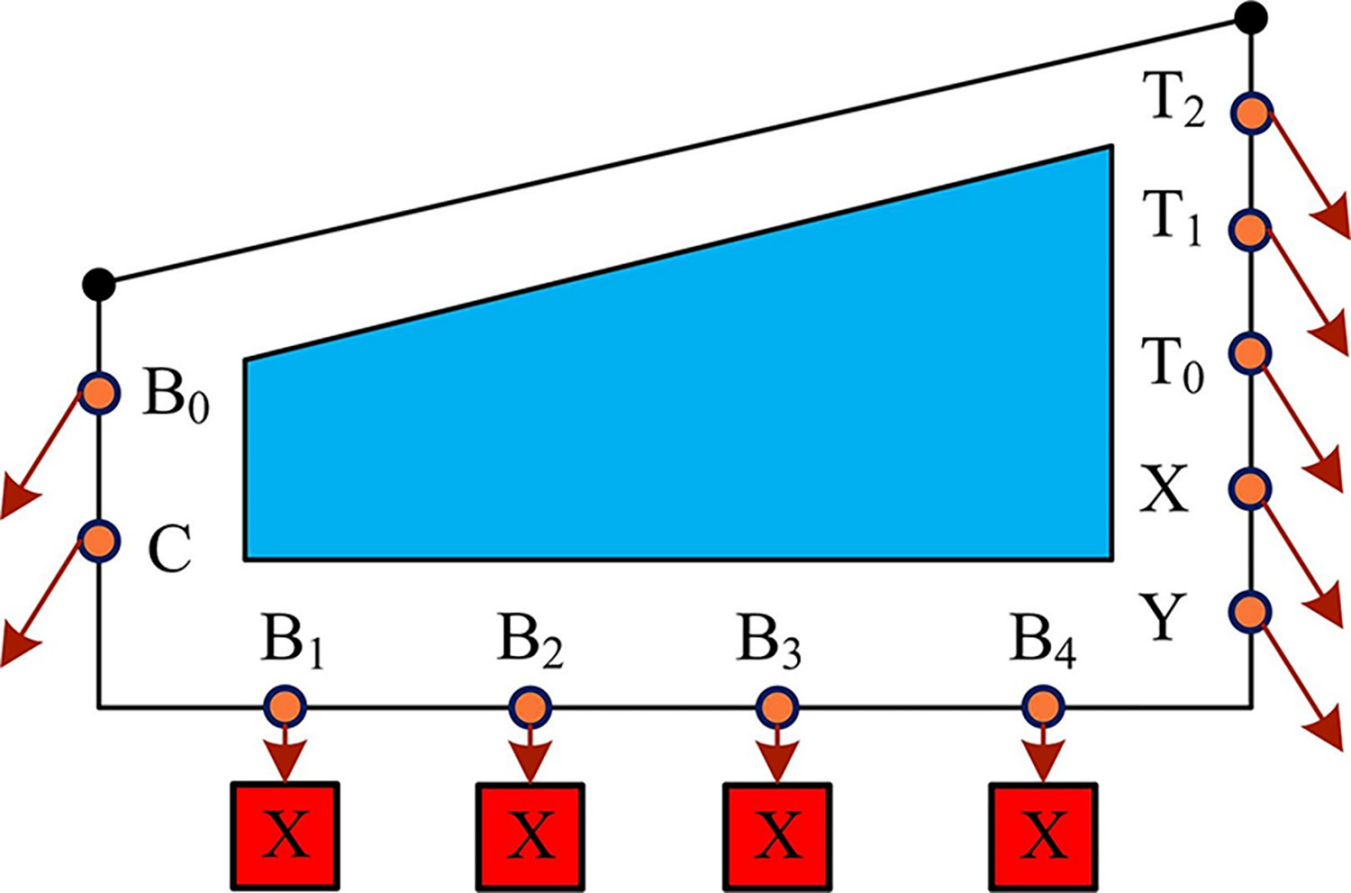
IBIO operator circuit.

The background of each frame is converted from the original image to 24 binary images. Only *X*^⊗5^ gate can be used to reverse the bit plane order, and the significant bit of the original background of each frame is inverted. Moreover, the lowest significant bit is the current highest significant bit. In summary, these three operators provide a full range of services for quantum video, from color transformation, frame sequence adjustment to encryption protection. The IBIO operator circuit is shown in Fig 6.

Fig 6 displays the circuit diagram for the IBIO operator. The video representation splits the original image into 24 binary images based on the bit plane. Therefore, reversing the order of bit planes only requires inverting the salient bits of each original frame, converting the lowest salient bit to the highest salient bit.

This unique design approach is a new exploration and breakthrough in quantum video processing technology, and plays an extremely important role in giving videos richer layers and expressive meanings.

The use of quantum representation for multi-media data, such as video, is necessary for the following reasons. Firstly, quantum systems can process information in parallel, which greatly improves processing efficiency. Secondly, by utilizing the superposition and entanglement properties of quantum systems, a large amount of data can be processed and stored simultaneously without increasing the complexity of the system.

### 3.3. Design of multimodal quantum color dynamic conversion and pixel-level mixing strategy

In the study of processing multi-media color information, the two QI processing operations constructed by the AMQRM model include color transformation operation and pixel blending operation. The color details of AMQRM color map are represented by Eq (12).


$$|M\rangle |C(M,Y,X)\rangle =|M_{1}M_{0}\rangle |C_{q-1}\cdots C_{0}\rangle$$ (12)

**Fig 7:**
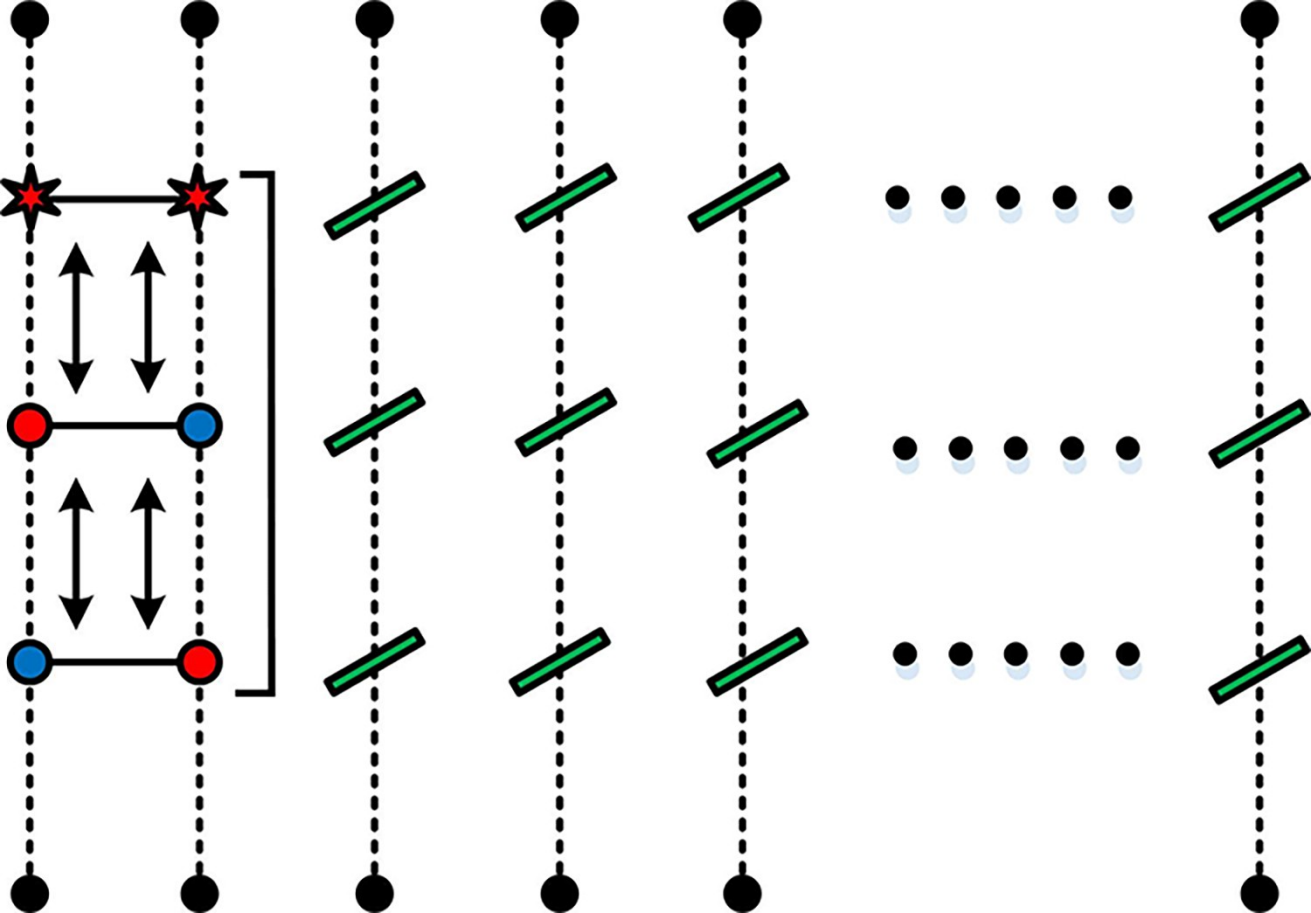
Tone transformation operator circuit.

In Eq (12), |*M*_1_*M*_0_〉 denotes the differentiated color channel and |*C*_*q*−1_⋯*C*_0_〉 denotes the image color change information. The color transformation operation has the capacity to alter the sequence of color priority display in the quantum graph. Primarily, it is essential to implement the adjustment of the RGB channel. The research design tone transformation operator (TTO) is capable of exchanging the R and G channel, B and G channel, as well as the R and B channel. The TTO line is shown in Fig 7.

Fig 7 shows the specific preparation circuit of a QRMW model for a color image with a size of 2×2 and an RGB three channel value range of [0,28–1].

The pixel color rearrangement operation of AMQRM color map requires image color mixing operator (ICSO), which can be divided into three kinds of operators, namely *ICSO*_*i*_, *ICSO*_*j*_, *ICSO*_*ij*_. *ICSO*_*i*_ as shown in Eq (13).


$$ICSO_{i}=\prod_{i=7,j=0}^{i=4,j=3}I^{⊗2n+2}⊗SWAP_{ij}⊗I^{⊗6}$$ (13)

In Eq (13), *SWAP* represents the quantum exchange operation. *ICSO*_*j*_ is shown in Eq (14).


$$ICSO_{j}=I^{⊗2n+2}⊗X^{⊗8}$$ (14)

*ICSO*_*ij*_ is shown as in Eq (15).


$$ICSO_{ij}\prod_{i=7,j=6}^{i=1,j=0}I^{⊗2n+2}⊗CNOT_{ij}⊗I^{⊗6}$$ (15)

**Fig 8:**
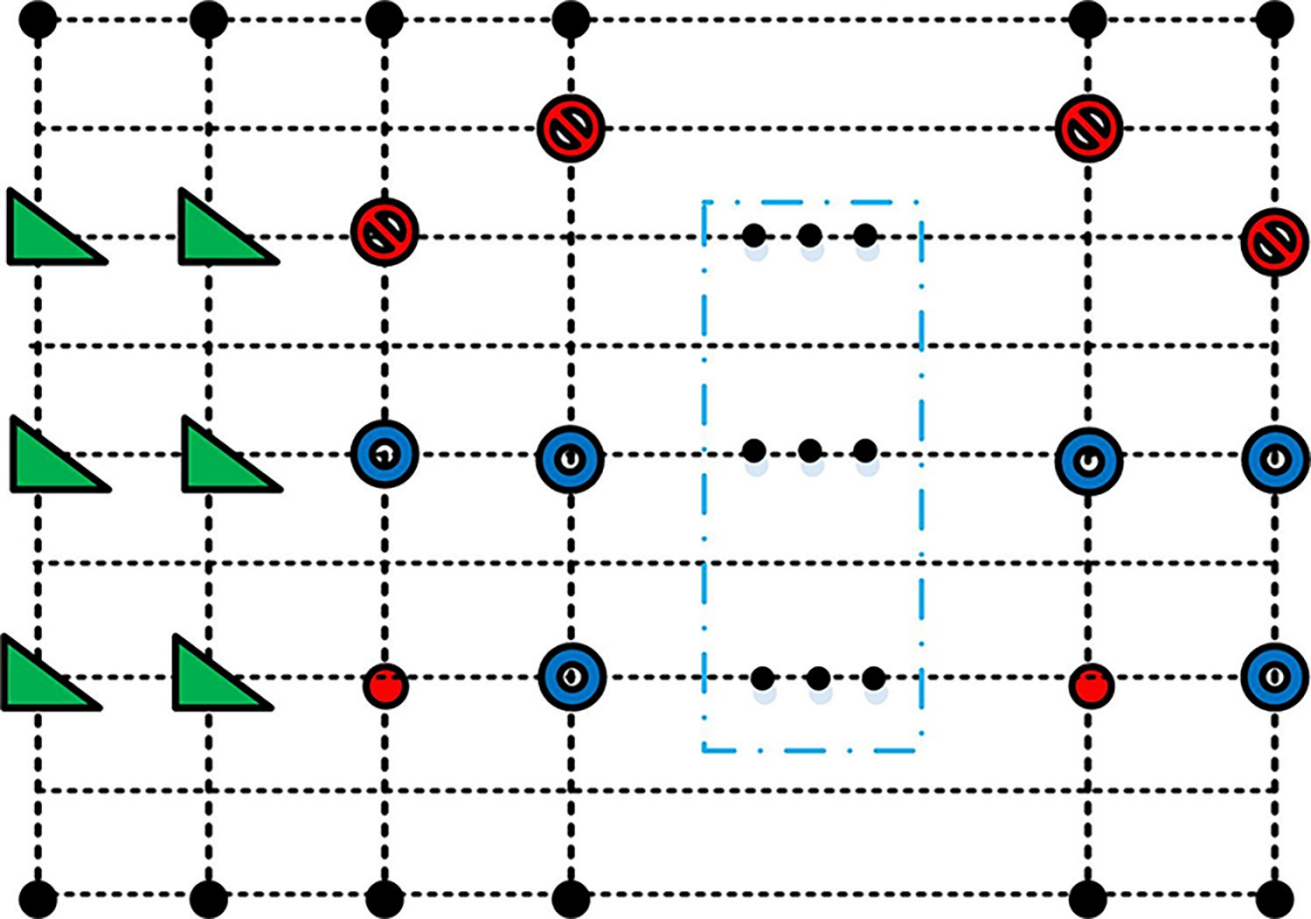
Image color scrambling operator circuit.

In Eq (15), *CONT* represents controlled ungated operation. The *ICSO*_*i*_ sub-operations mainly enable the exchange of low and high quantum bits in a sequence of quantum bits displaying color information. The ICSO line is shown in Fig 8.

Fig 8 displays the simplified quantum circuit, which greatly reduces its complexity. The preparation line route has been reduced from 48 4-CNOT gates to 16 4-CNOT gates and 16 3-CNOT gates. The *ICSO*_*i*_ suboperation performs an *X* gate on the sequence of quantum bits holding the color information, allowing all the original information to be reversed. Finally, the *ICSO*_*j*_ sub-operation performs a *CONT*-gate on the sequence of quantum bits holding the color information from high to low. In this way, *ICSO*_*i*_ operation, *ICSO*_*i*_ operation and *ICSO*_*j*_ operation are applied to the color image, and the colors of the image are rearranged. The pixel color rearrangement operation, based on the AMQRM architecture, is highly effective in concealing the original features and information of the image. The visual effect is that the rearranged image contains minimal resemblance to the original. In the real world, the study applies this color re-arrangement operation to medical images. The algorithm is highly applicable to both color and grayscale images and visually hides the original data, which shows excellent cryptographic performance. In conclusion, these two QI processing operations based on the AMQRM model, the color transformation operation and the pixel mixing operation, not only demonstrate their unique concepts and superiority theoretically, but also show their high performance and broad development prospects in practical applications.

As a key element in multi-media content processing, multi-media color information can be encrypted by various POs designed to achieve copyright protection without compromising the quality of the original content information. Unlike traditional image color encryption, the image color compensation operator is implemented through quantum bit inversion, which can greatly change the color tone of the original multi-media content, thereby enhancing the expression effect of multi-media content, and the color tone change also plays a role in copyright protection. The frame shifting operator adjusts the playback order by changing the time index of two frames, thereby changing the expression of multi-media content to achieve content encryption. Finally, a bit plane inversion operator is used to invert the entire bit plane, achieving simple multi-media content encryption and protecting the copyright of the original content.

In the context of research design, the fundamental structure of quantum video is a sequence of frames that are not simply conventional images. Instead, these frames are quantum states encoded using the principles of quantum mechanics. Each frame of the image is processed in parallel using quantum bits, overcoming the limitations of traditional multi-media processing. The model uses quantum state superposition to play back time-stamped photos sequentially, improving the speed and efficiency of information processing.

In terms of multi-media, the research and design of a multi-media quantum RM can present videos in the form of quantum bitmaps, which can process a large amount of image data at once and improve processing efficiency. The model is based on the BRQI model and uses a decomposition process that generates 24 black-and-white images from a single frame of color photographs. This process produces a set of coordinate data for each pixel, along with bit-plane sequence data and the black-and-white data associated with each pixel within the frame.

In terms of operation operators, research is conducted on the use of image color compensation operators to invert color qubits, thereby achieving quantum video tone adjustment. By using the IFDO to change the playback order of any frame in the video, the effect of adjusting the playback order can be achieved. To achieve video encryption, the study uses the image bit plane inversion operator to transform the background of each frame from the original image into 24 binary images. Then, the bit plane order is reversed by using gates.

## 4. Performance examination of a quantum operator video representation framework

In the past series of tests, the research conducts a detailed performance examination on quantum’s video representation framework. First, the data performance of the framework under different resolution scenarios is summarized in terms of space overhead and time complexity. After that, it tests the PO effect under different data-sets, and the image security under color operator processing.

### 4.1. Framework overhead analysis

A series of software and hardware are used in the simulation process, as shown in Table 2.

**Table 2:** Device table.

Serial number	Device name	Describe
1	Computer hardware	High performance workstation equipped with Intel Core i9 processor, 64GB RAM, NVIDIA RTX 3080 graphics card
2	Quantum simulator	IBM Qiskit Aer Simulator
3	Storage device	2TB NVMe SSD storage
4	Programming interface	Qiskit Quantum Programming Framework
5	Operating system	Ubuntu 20.04 LTS
6	Monitor	27 inch 4K resolution monitor
7	Network environment	Gigabit Ethernet connection
8	Image processing tools	OpenCV library
9	Data set	Medical image dataset, Lena image dataset (32x32 size)
10	Programming language	Python 3.8

In Table 2, the initial step is to create a quantum circuit that includes the definition of quantum bits and classical bits, as well as the initialization of quantum states. Furthermore, the definition of various quantum operations based on this structure must be provided. Tone conversion operations can be achieved through a series of CNOT gates to exchange different color channels. Quantum gates correspond to mathematical transformations and act on the states of quantum bits. Then it is necessary to compile the subcircuits, optimize the quantum circuits, reduce the number of quantum gates, and convert the quantum circuits into low-level instructions suitable for execution on IBM quantum computers. The hardware precisely mimics the behavior of quantum computers, performing defined quantum operations and returning measurement results of quantum states. Finally, the results are stored in classical bits for easy extraction and analysis. The study first prepares 4 frames of 2 × 2 sized video samples. The specific sample situation is shown in Table 3.

**Table 3:** Frame video example.

Frame	Pixel color	
Frame 1	Pixel (0,0)	Red
	Pixel (0,1)	Green
	Pixel (1,0)	Blue
	Pixel (1,1)	Yellow
Frame 2	Pixel (0,0)	Cyan
	Pixel (0,1)	Magenta
	Pixel (1,0)	White
	Pixel (1,1)	Black
Frame 3	Pixel (0,0)	Blue
	Pixel (0,1)	Red
	Pixel (1,0)	Green
	Pixel (1,1)	Yellow
Frame 4	Pixel (0,0)	Black
	Pixel (0,1)	White
	Pixel (1,0)	Magenta
	Pixel (1,1)	Cyan

Based on Table 3, when preparing 4 frames of 2×2 sized video samples, the first step is to initialize a register containing 10 qubits. It is essential to assign the qubits within the register to the specific location of each pixel, as well as the bit plane sequence data, the pixel binary data, and the video frame information. This is done for the purpose of information storage. The quantum register must be initialized to an all-zero state, and then the initial quantum state must be converted into an intermediate quantum state through the application of quantum operators. The intermediate state needs to contain pixel position information, time information, and bit plane information. Finally, the color information is written frame by frame into the intermediate quantum state to prepare the video samples for sampling.

The study conducts separate experiments on framework performance overhead and operator effectiveness. The experimental environment information is shown in Table 4.

**Table 4:** Experimental setup.

Classification	Performance cost test environment	Experimental environment for processing operators and color operators
Experimental platform	Desktop computer with IBM Qiskit installed	Desktop computer with IBM Qiskit installed
CPU	Intel i7-8809G (4 cores/8 threads)	AMD Zen3 5800X (8 cores/16 threads)
Memory	32GB DDR4-3200	32GB DDR4-3200
Python version	3.8	3.8
Qiskit version	0.33.1	0.33.1

As shown in Table 4, During the experiment, a quantum circuit has been established using the Qiskit interface and Python language to implement a QRMW model image of size 2×2. The collapse process is measured and restored to a classical image. Medical images and Lena images with a size of 32 ×32 are generated on this basis. Moreover, the color transformation operations are performed on the Lena image quantum circuit to construct the quantum circuit. After running, the collapse measurement is performed to restore the object to its original shape. Then, a scrambling operator route is constructed based on medical and Lena images to continue the running, collapse measurement, and restoration process. During experiments on POs, the original video is divided into 64 groups of 8 frames of 32 × 32 video blocks. The 8 frames of 32 × 32 video blocks are generated from a single preparation. Finally, the 64 sets of video fast data are carefully pieced together.

This study analyzes the performance of each quantum video representation framework in terms of grayscale space cost, chromaticity space cost, and time complexity in different scenarios, based on the simulated data table generated before the study. The author has verified the method and data used in this article for repeated experiments, making it easier for other researchers to understand and apply. This study selects data-sets from different resolution scenes, including low resolution, medium resolution, and high-resolution scenes, to comprehensively cover common multi-media data usage. In terms of data processing, it follows the general principles and good practices in the field of quantum information science. A set of POs, including image complement operator, bit plane inversion operator, and frame shift operator, are constructed around advanced multi-media quantum RMs. When analyzing data, list the data in sequence, explain their meanings, and compare the results of different models. The comparison of quantum video representation framework operations is shown in the Table 5.

**Table 5:** Comparison of framework operations.

Quantum video representation framework	Pedagogical operation	References
FFQM (full frequency quantum model)	Using a full frequency quantum model for video representation, encoding through probability amplitude, suitable for grayscale space.	X. Han et al. [42]
MFQM (multi-frequency quantum model)	Using a multi frequency quantum model to represent videos, encoding through probabilistic amplitude, and focusing on color space processing.	B. Zhu, et al. [43]
QVNEQR (quantum video non-equilibrium quantum representation)	Adopting quantum non-equilibrium representation and encoding based on fundamental states, suitable for grayscale space representation.	C. Hou, et al. [44]
QVNCQI (quantum video non-equilibrium color quantum information)	Using the quantum non-equilibrium color quantum information model, encoding through basic states is applicable for color space representation.	J. Vodeb, et al. [45]
AMQRM (adaptive multi-quantum representation model)	Adopting an adaptive multi quantum representation model, combined with grayscale and color space, encoding through basic states to achieve flexible quantum video representation.	This study

From Table 5, the study mainly uses the UCF-101 dataset in the experiment, which is a publicly available dataset from the University of Central Florida. The dataset contains a total of 13320 videos and 101 categories. This dataset is suitable for action recognition testing of quantum video processing frameworks. The richness of its collection of actions and scene changes makes it suitable for the evaluation of performance at different resolutions and for the analysis of frame processing operators. The dataset link is https://tianchi.aliyun.com/dataset/92158. Frame overhead is illustrated in Table 6.

**Table 6:** Comparison of quantum video representation frameworks at 2^m^2^2n^ pixel scale.

Presentation framework	Gray space overhead	Color space overhead	Time complexity	Encoding method
FFQM (full frequency quantum model)	2n+m+2	\	24(n+m)	Probability amplitude
MFQM (multi-frequency quantum model)	\	2n+m+4	24×24(n+m)	Probability amplitude
QVNEQR (quantum video non-equilibrium quantum representation)	2n+m+8	\	16(n+m)×22(n+m)	Fundamental state
QVNCQI (quantum video non-equilibrium color quantum information)	\	2n+m+24	48(n+m)×22(n+m)	Fundamental state
AMQRM (adaptive multi-quantum representation model)	2n+m+4	2n+m+8	64(n+m)×22(n+m)	Fundamental state

In Table 6, when comparing different quantum video representation frameworks, I mainly consider four aspects: spatial overhead (gray scale and color scale), time complexity, and coding method. In reality, although increasing the resolution can improve the video quality, it also increases the computational complexity and spatial overhead. When selecting a framework for quantum video representation, it is important to balance time complexity and space overhead based on the specific situation. Among the four aspects compared, AMQRM demonstrates strong performance in terms of storage space overhead and time complexity. Additionally, it utilizes the ground state coding method, which further enhances its superiority. Firstly, in the grayscale space overhead, FFQM uses 2n+m+2 space, while QVNEQR uses 2n+m+8 space. AMQRM, on the other hand, consumes only 2n+m+4 space, which is 0.9 more than the FFQM control and even more than the QVNEQR, which saves 3.4 space. This is significant given that 2^m^2^2n^ pixel scale videos are in quantum storage devices. This shows that AMQRM has a significant advantage in grayscale space overhead. In terms of color space overhead, both MFQM and QVNCQI consume more space than AMQRM’s 2n+m+8, and AMQRM shows superior space efficiency among the four frames compared. In terms of time complexity, AMQRM only takes 64(n+m)×22(n+m), which is less than the time consumed by other frameworks. In terms of coding method, AMQRM adopts the base state coding method, which has better efficiency and stability in dealing with large-scale video data than the probabilistic amplitude coding method used by other frameworks. Overall, combining the space overhead, time complexity, and coding method, AMQRM has a significant advantage of saving space and reducing time complexity when processing 2^m^2^2n^ pixel scale video, and crucially, the coding method is stable. The results of the comparative analysis presented above lead to the conclusion that AMQRM is an efficient, resource-saving, stable, and high-quality choice in the quantum video representation framework, particularly suited to the processing of large-scale video data. The specific performance calculation data is shown in Table 7.

**Table 7:** Performance comparison under different dimensions.

Scene	Presentation framework	Gray space overhead	Color space overhead	*p*-value	Time complexity	Encoding strategy
Low resolution (n = 4, m = 3)	FFQM	12.7	\	\	O(2^5^)	Probability amplitude
	MFQM	\	15.9	\	O(2^8^)	Probability amplitude
	QVNEQR	19.2	\	\	O(2^5^)	Fundamental state
	QVNCQI	\	28.3	\	O(2^4^)	Fundamental state
	AMQRM	15.8	19.5	0.005	O(2^6^)	Fundamental state
Medium resolution (n = 6, m = 3)	FFQM	17.7	\	\	O(2^8^)	Probability amplitude
	MFQM	\	21.9	\	O(2^12^)	Probability amplitude
	QVNEQR	27.2	\	\	O(2^8^)	Fundamental state
	QVNCQI	\	36.3	\	O(2^7^)	Fundamental state
	AMQRM	23.8	27.5	0.007	O(2^9^)	Fundamental state
High resolution (n = 10, m = 4)	FFQM	23.7	\	\	O(2^13^)	Probability amplitude
	MFQM	\	26.9	\	O(2^20^)	Probability amplitude
	QVNEQR	37.2	\	\	O(2^13^)	Fundamental state
	QVNCQI	\	47.3	\	O(2^13^)	Fundamental state
	AMQRM	33.8	40.5	0.004	O(2^14^)	Fundamental state

**Fig 9:**
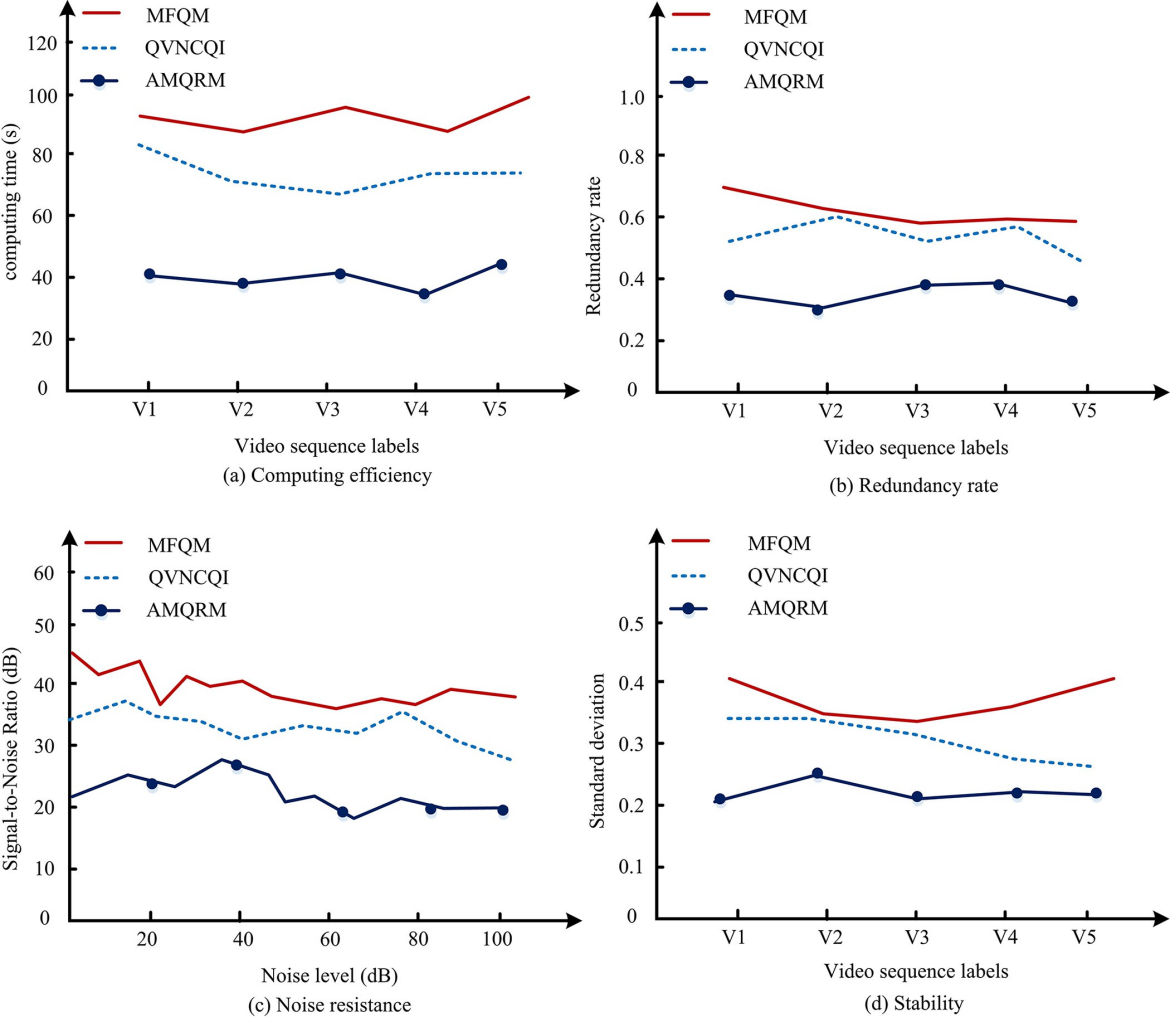
Model performance comparison.

As shown in Table 7, the performance of each quantum video representation framework varies in different scenarios, and in general, AMQRM maintains relatively good performance in all cases. Compared to the other frameworks, AMQRM consistently shows advantages in terms of space overhead as well as time complexity in low, medium, and high resolution scenarios. Considering first the low resolution scenario (n = 4, m = 3), which is a more basic video test criterion, in this case AMQRM has a grayscale overhead of 15.8, a color overhead of 19.5, and a time complexity of O (2^6^). Compared to the other models, both FFQM and QVNEQR perform likewise in terms of grayscale overhead, but are not dominant for processing efficiency because of higher time complexity. Both MFQM and QVNCQI demonstrate satisfactory performance with regard to color overhead. However, they exhibit a higher time complexity. In medium resolution scenarios (n = 6, m = 3), AMQRM is simply the shining star. Its grayscale overhead and color overhead are 23.8 and 27.5, respectively, and its time complexity29 is at a good level. The other frameworks fall slightly short compared to AMQRM in terms of consumption and computational complexity. The problem also becomes more complex when raised to high resolution scenes (n = 10, m = 4). At this point, AMQRM with a grayscale overhead of 33.8 still continues to perform well. Even though the time complexity is increased to 2^14^, it still outperforms the other models, especially in the processing of color scale, which reaches 40.5 is appreciated. In summary, all aspects of the analysis and data show that the AMQRM framework is at the forefront of superiority and applicability in handling quantum video tasks in different scenarios. Of course, despite the overall excellent performance, it is recommended to select and apply it according to the specific situation in order to get the optimal solution. According to the data in Tables 1 and 2, due to the similar performance of FFQM and QVNEQR in grayscale overhead, the processing efficiency is poor when the time complexity is high, and MFQM and QVNCQI have high overhead. Therefore, the other three models with better overall performance are selected for subsequent experiments, as shown in Fig 9.

From Fig 9A, the AMQRM model has the shortest overall computation time, which is around 40s. From Fig 9B, the AMQRM model has the lowest redundancy rate fold position, which is around 0.37. From Fig 9C, the AMQRM model has the best signal-to-noise ratio, which fluctuates but has the lowest position. From Fig 9D, the standard deviation of the AMQRM model is generally at the minimum position and the model is the most stable.

### 4.2. Comparison of the effects of processing operators

The study is conducted to compare the PO effects by setting the data set levels with different quantum bit numbers and testing the third operator separately. The computational results of the framework used for the comparison are shown in Table 8.

**Table 8:** Compare the calculation results of the framework used.

Differentiation calculation	Data-set (quantum bit count)	Framework	ICCO operator	IBIO operator	IFDO operator	Total
	10	FFQM	10000δ	10000δ	24000δ	44000δ
	10	MFQM	10000δ	10000δ	24000δ	44000δ
	10	QVNEQR	880	120	24000δ	25000δ+1000
	20	FFQM	20000δ	20000δ	54000δ	94000δ
	20	MFQM	20000δ	20000δ	54000δ	94000δ
	20	QVNEQR	1760	240	54000δ	56000δ+2000
	50	FFQM	50000δ	50000δ	144000δ	244000δ
	50	MFQM	50000δ	50000δ	144000δ	244000δ
	50	QVNEQR	4400	600	144000δ	148000δ+5000
	100	FFQM	100000δ	100000δ	294000δ	494000δ
	100	MFQM	100000δ	100000δ	294000δ	494000δ
	100	QVNEQR	8800	1200	294000δ	302000δ+10000
Aggregation	Framework		Overhead			
	FFQM		1132000δ			
	MFQM		1132000δ			
	QVNEQR		1260000δ + 18000			

As shown in Table 8, the δ represents qubits. The overhead of the FFQM and MFQM frameworks increases linearly with the number of quantum bits in the data-set. This is due to the fact that, for both frameworks, all the operators need to operate on each pixel, so the overhead of these frameworks increases linearly as the number of pixels increases (i.e., the number of quantum bits increases). For example, for the FFQM framework, when the number of quantum bits doubles, the overhead also doubles. This is due to the large number of operations required by this framework for both the implementation of the ICCO operator and the IBIO operator. The overhead growth trend of the QVNEQR framework is different from that of FFQM and MFQM. The overhead of the ICCO and IBIO operators grows linearly with the number of quantum bits in the data-set, but the growth is smaller compared to the FFQM and MFQM frameworks. This is due to the fact that the ICCO and IBIO operators only require 8 quantum bits to represent color information. Therefore, even if the number of pixels increases, the overhead of the ICCO and IBIO operators does not increase significantly. However, for the IFDO operator, the overhead of the QVNEQR framework still grows linearly with the increase in data-set and pixels. For example, when the data-set increases from 10 to 100, the overhead of the IFDO operator increases approximately 12 times. Although the overhead of the FFQM and MFQM frameworks increases linearly with the increase in data-set size, they always have a smaller overhead than the QVNEQR framework. This suggests that the FFQM and MFQM frameworks may be more advantageous when dealing with large-scale data-sets. The overhead of the QVNEQR framework is greater than that of the FFQM and MFQM frameworks on all the passed data-sets. Although for ICCO operator and IBIO operator, this framework is more efficient than the other frameworks, but for IFDO operator, it has more overhead. The detection results of the three operators under AMQRM are shown in Table 9.

**Table 9:** Three operator detection results under AMQRM.

Differentiation calculation	Data-set (color qubits/pixel qubits)	Framework	ICCO operator	IBIO operator	IFDO operator	Total
	10/10	AMQRM	8δ	12	39δ	47δ + 12
	20 / 20		16δ	15δ	87δ	118δ
	50 / 50		40δ	35δ	216δ	291δ
	100 / 100		80δ	65δ	429δ	574δ
Aggregation	Framework		Overhead			
	AMQRM		1060δ + 12			

In Table 9, the δ represents qubits. The performance of the AMQRM quantum video representation framework is comprehensively performance examined and compared, especially with the application of three quantum operators, ICCO, IBIO and IFDO. According to the data, AMQRM exhibits stable performance regardless of the change in the size (number of quantum bits) of the data-set. In the 10/10 data-set, the overheads of using the ICCO, IBIO, and IFDO operators, respectively, are 8 δ, 12, and 39 δ, for a total of 59 δ + 12. However, as the data-set size grows, the overheads of all three operators increase. In the 20/20 data-set, the overhead of the ICCO, IBIO and IFDO operators grows to 16δ, 15δ and 87δ, respectively, and the total overhead grows to 118δ. This shows that as the data-set size grows, the overhead of the whole system grows linearly. Further increasing the data-set, when the data-set size is 50/50, the overheads of ICCO, IBIO, and IFDO operators reach 40δ, 35δ, and 216δ, respectively, and the total overhead reaches 291δ. Finally, in the 100/100 data-set, the overheads of the three operators climb further, rising to 80δ, 65δ, and 429δ, respectively, and the total overhead reaches 574δ. Note that δ in the table indicates the unit of measure of overhead. According to the tabular data, the overheads of all three operators, ICCO, IBIO and IFDO, on the AMQRM framework increase linearly with the increase of the number of color quantum bits/pixel quantum bits in the data-set. The discovery of this pattern provides an important reference for predicting the computational complexity and resource requirements of large-scale quantum video processing tasks. In conclusion, the stability and predictability of computational complexity of the AMQRM framework in processing different quantum video tasks enable it to show good performance when facing different sized data-sets. This indicates that the AMQRM framework is designed with scalability in mind and is capable of handling quantum video processing tasks of various sizes in practical applications.

### 4.3. Comparison of color operator effects

**Fig 10:**
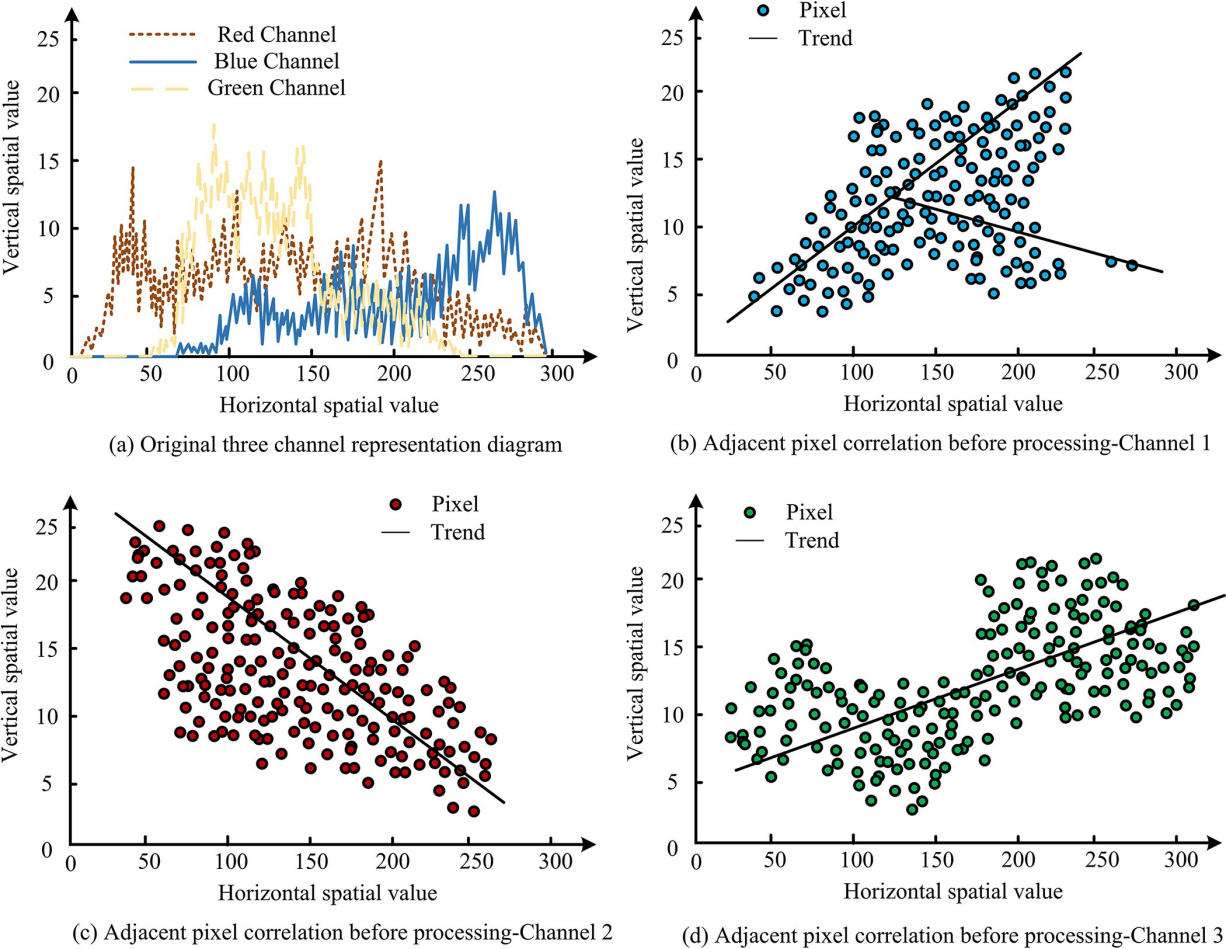
Image color scrambling operator circuit.

When comparing the effect of color operator, different dimensions will be analyzed from the RGB three channels with horizontal axis, vertical axis or diagonal axis, respectively. The condition of the image before processing is shown in Fig 10.

**Fig 11:**
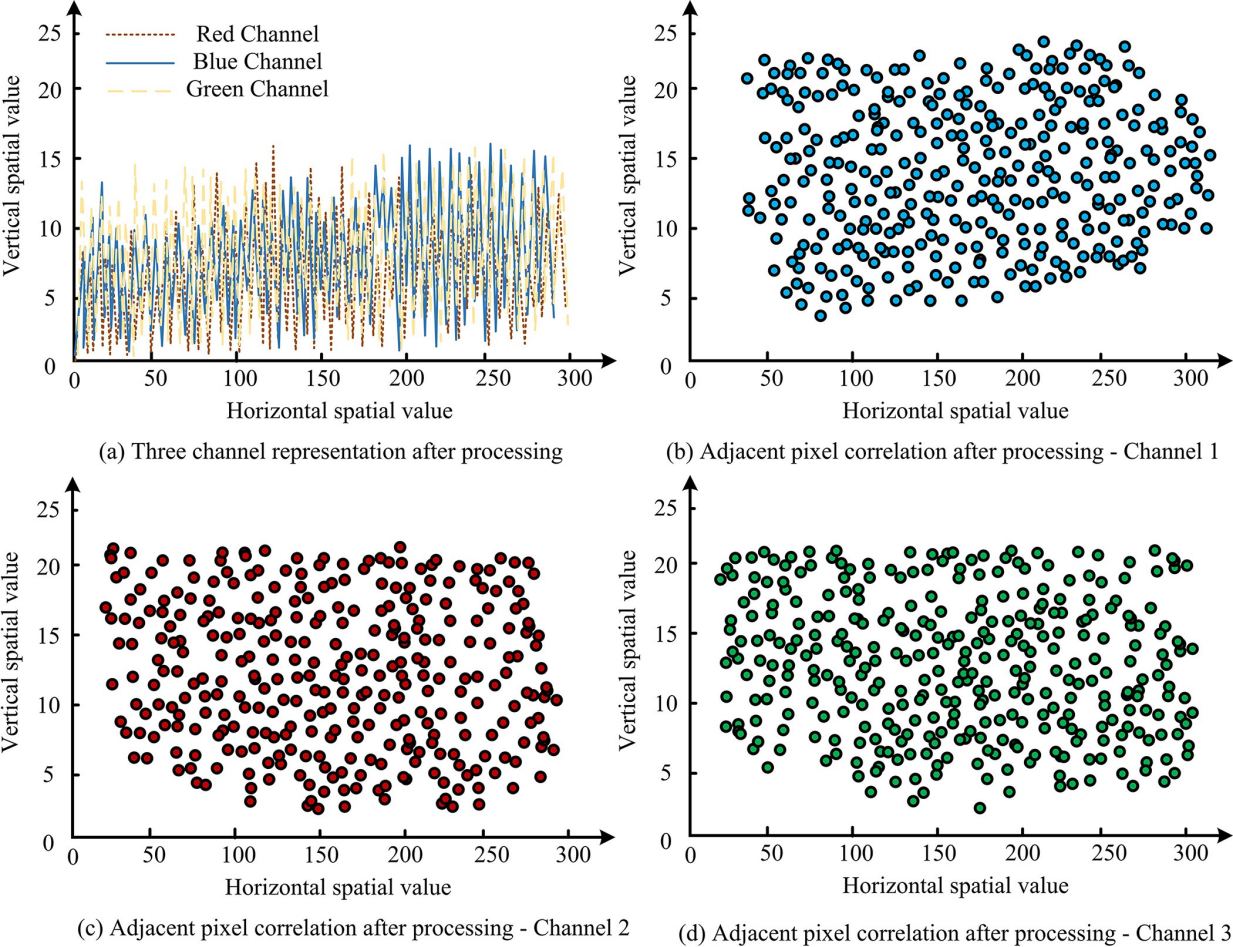
Image color scrambling operator circuit.

In Fig 10, the red channel, green channel and blue channel are the three branches of the RGB channel, the histogram of the original image shows the distribution of the image elements in the three RGB channels, and the original distribution is uneven and distinctive. However, after applying the research method, the image elements achieve a balanced distribution over the whole range, which in turn hides the details of the original image element distribution and facilitates the encryption of the image, as shown in Fig 11.

**Fig 12:**
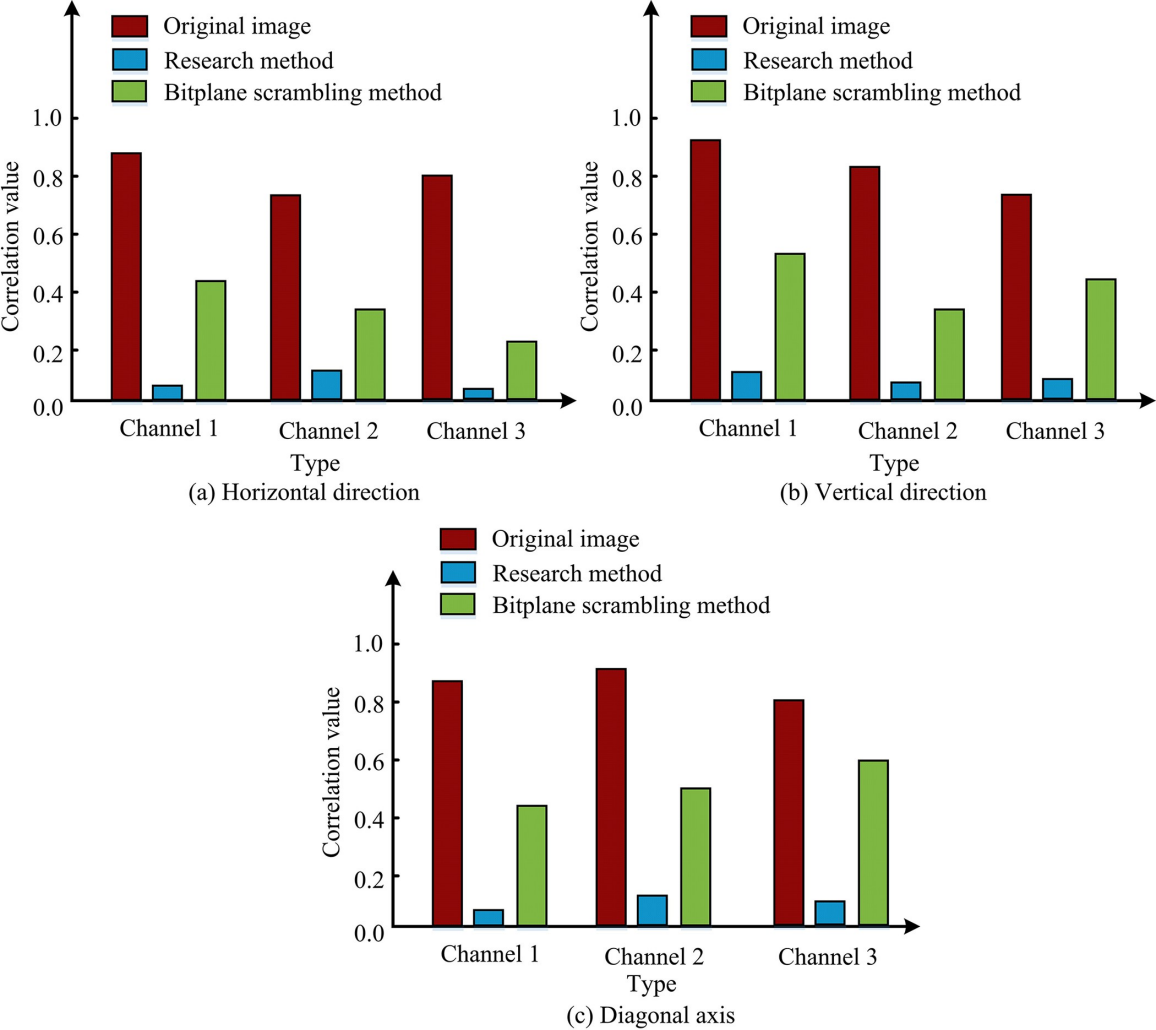
Image color scrambling operator circuit.

Fig 11 shows that the distribution of image elements in the original image is not balanced across the three RGB channels, with a clear distribution pattern. However, the research method used to adjust the image results in a well-balanced distribution of image elements across all channels, covering almost the entire range. This change significantly improves the image’s security level, although it does obscure some of the original image element distribution details. The effect of image disorganization is shown in Fig 12.

In Fig 12, the correlation coefficients of the images, both in the horizontal, vertical and diagonal axes, are significantly reduced under the guidance and effect of the research design method, and in some cases, these coefficients are even close to zero. This shows that the correlation between the original image and the moved image in each color channel is drastically diminished thus showing that the research design operator can effectively change various properties of the image including color, texture, and so on. In this scenario, the quantum effect primarily weakens the proximity of the image elements, altering the interrelationships between them and rendering the individual elements more independent. This outcome is highly advantageous for information hiding, as the main information of the original image can be successfully concealed within an image that appears unrelated to the original. This approach is not only efficient, but also hard to detect. When seeing the moved image, it is almost impossible to detect the hidden information in it, which achieves the goal: to achieve information steganography without destroying the original image.

Overall, in the comparative analysis of framework overhead, the AMQRM model designed in this study demonstrates significant advantages. The model demonstrates robust performance in both grayscale and color spaces, providing a more space-efficient alternative to existing models. It also has a significant advantage in terms of time complexity, with reduced overall computational overhead, accelerated computational speed, and improved computational efficiency. Whether it is simple video inspection or high-resolution video processing, AMQRM can provide stable and efficient performance. In addition, due to the ground state encoding method used in the AMQRM model, it is more efficient and stable compared to the probability amplitude encoding method. In the context of large-scale data processing, the advantages of AMQRM in terms of storage space, computational overhead, and computation time will be particularly pronounced. Therefore, the choice of application scenarios for the AMQRM model is crucial, as appropriate scenarios can enhance its effectiveness. In future quantum video processing, the AMQRM model will undoubtedly provide an efficient and stable solution. The horizontal comparison of different models are shown in Table 10.

**Table 10:** Horizontal comparison.

Model	Processing speed (frames per second)	Data processing capacity (MB)
AMQRM	1000	5000
Quantum enhanced video processing model (QEVPM)	850	4500
Neural network-based video optimization model (NNBVOM)	900	4800
Efficient video compression and processing model (EVCPM)	920	4700

Table 10 horizontally compares the performance of processing speed and data processing capability. The calculation method for processing speed is the number of frames processed per second, which is used to measure the latency and smoothness during video processing. The calculation method for data processing capacity is defined as the amount of data that can be processed per second. This metric is utilized to assess the stability and efficiency of the model when processing high-resolution and large data videos. The AMQRM model has advantages in processing speed and data processing capacity compared to other models. In particular, the AMQRM model has a processing speed of 1000 frames per second and a data processing capacity of 5000MB, which is then followed by the QEVPM model with a processing speed of 850 frames per second and a data processing capacity of 4500MB. The NNBVOM model and EVCPM model have relatively lower processing speeds. In summary, in the horizontal comparison, the AMQRM model has higher processing efficiency and stability. The verification results of the application effects of image color compensation operator, frame displacement operator, and bit plane inversion operator are shown in Table 11.

**Table 11:** Horizontal comparison.

Frame sequence number	Frame displacement	Pixel coordinates	Original color value	Color Supplement	Reverse the position plane	Color shuffling results
Frame 1	Frame 4	(0,0)	(120, 200, 150)	(135, 55, 105)	(240, 155, 90)	(210, 70, 95)
Frame 1	Frame 3	(0,1)	(255, 100, 50)	(0, 155, 205)	(55, 200, 105)	(10, 140, 245)
Frame 2	Frame 2	(1,0)	(75, 50, 180)	(180, 205, 75)	(30, 205, 135)	(170, 220, 45)
Frame 2	Frame 1	(1,1)	(90, 230, 60)	(165, 25, 195)	(70, 195, 30)	(125, 45, 225)
Frame 3	Frame 1	(0,0)	(15, 45, 230)	(240, 210, 25)	(200, 125, 135)	(150, 175, 15)
Frame 3	Frame 2	(0,1)	(210, 160, 75)	(45, 95, 180)	(190, 110, 85)	(220, 80, 145)
Frame 4	Frame 4	(1,0)	(255, 180, 90)	(0, 75, 165)	(125, 210, 30)	(235, 115, 95)
Frame 4	Frame 3	(1,1)	(100, 200, 40)	(155, 55, 215)	(80, 145, 220)	(40, 255, 85)

As shown in Table 11, firstly, the ICCO operator has excellent pixel color reversal ability, where the original color of frame 1 pixel is (120, 200, 150). It is transformed into (135, 55, 105) after operator reversal. It is indicated that the ICCO operator can effectively achieve fast color conversion by setting inverse qubits. In terms of frame displacement operator, the pixels in frame 1 are converted into frames 4 and 3 after displacement operation. Similarly, frames 2, 3, and 4 all demonstrate substantial displacement, indicating that the sequence of frames in the video has undergone notable alterations and that the expression of the original video content has been significantly transformed. In terms of IBIO operator, the color of frame 1 (0,0) pixels changes from (120, 200, 150) to (210, 70, 95), and other frames also experience significant color changes. Therefore, color scrambling can successfully achieve image encryption and hide image information. Three operators can effectively achieve image color, image expression, and image encryption, which provides a technical foundation for quantum video processing.

## 5. Conclusion

Traditional video processing methods are often limited in processing speed and efficiency when faced with increasingly complex application scenarios. Accordingly, a high-efficiency and low-power quantum video display framework was developed to improve the efficiency of multi-media quantum processing. This framework was based on quantum operator processing technology and included a series of POs such as image color compensation operator, bit plane reversal operator, and frame shift operator. To further address image security issues, the study also introduced color transformation operations and pixel blending operations. To test the effectiveness of the model, the framework overhead and operator effects were analyzed in different scenarios. The results showed that in high-resolution scenes (n = 10, m = 4), the grayscale overhead of AMQRM reached 33.8, the chroma reached 40.5, and the time complexity was 214. For medium resolution scenes (n = 6, m = 3), the grayscale and chroma overheads were 23.8 and 27.5, respectively. For low resolution scenes (n = 4, m = 3), the grayscale and chroma overheads were 15.8 and 19.5, respectively. When testing the effectiveness of the operators, on the 100/100 dataset, the overhead of the three operators was 80 δ, 65 δ, and 429 δ, respectively, with a total overhead of 574 δ. In addition, the element distribution of the three RGB channels in the image processed by the color operator was more balanced. Furthermore, the original image’s pixel distribution details have been effectively concealed, thereby markedly enhancing the image’s security. The design of this framework exhibited notable advantages in processing efficiency and cost control, while also demonstrating a balanced distribution of RGB channel elements in color processing, thereby enhancing image security. The technology has a variety of potential applications, including intelligent monitoring and military encryption. Although the research and design model has considerable practical significance, it is a universal model that may not be able to meet some specific image processing requirements. It is therefore recommended that future research be directed towards the development of a modular design that can be adapted to meet specific needs.

## Supporting information

S1 Dataset (DOCX)
