# Supplementary material for: Representation models and processing operators for quantum informational multi-media
Source: PLoS One. 2025 Jan 16;20(1):e0313294. doi: 10.1371/journal.pone.0313294 (PMC11737787; doi:10.1371/journal.pone.0313294)
Supplement: S1 Dataset — (DOCX) [file pone.0313294.s001.docx]

The data in Figure 9

| Computing time (s) | | | |
| --- | --- | --- | --- |
| Video sequence labels | MFQM | QVNCQI | AMQRM |
| V1 | 95 | 83 | 41 |
| V2 | 84 | 72 | 40 |
| V3 | 101 | 68 | 43 |
| V4 | 86 | 78 | 37 |
| V5 | 104 | 79 | 48 |
| Redundancy rate | | | |
| Video sequence labels | MFQM | QVNCQI | AMQRM |
| V1 | 0.73 | 0.38 | 0.37 |
| V2 | 0.62 | 0.6 | 0.31 |
| V3 | 0.6 | 0.55 | 0.421 |
| V4 | 0.61 | 0.59 | 0.42 |
| V5 | 0.62 | 0.48 | 0.38 |
| Signal-to-Noise Ratio (dB) | | | |
| Noise level (dB) | MFQM | QVNCQI | AMQRM |
| 20 | 35 | 34 | 24 |
| 40 | 42 | 31 | 27 |
| 60 | 36 | 32 | 22 |
| 80 | 35 | 37 | 20 |
| 100 | 39 | 30 | 21 |
| Standard deviation | | | |
| Video sequence labels | MFQM | QVNCQI | AMQRM |
| V1 | 0.41 | 0.34 | 0.21 |
| V2 | 0.35 | 0.33 | 0.26 |
| V3 | 0.33 | 0.31 | 0.22 |
| V4 | 0.36 | 0.27 | 0.23 |
| V5 | 0.42 | 0.28 | 0.22 |

The data in Figure 12

| Horizontal direction | Type | Channel 1 | Channel 2 | Channel 3 |
| --- | --- | --- | --- | --- |
|  | Original image | 0.88 | 0.77 | 0.82 |
|  | Research method | 0.12 | 0.13 | 0.04 |
|  | Bitplane scrambling method | 0.41 | 0.38 | 0.24 |
| Vertical direction | Original image | 0.93 | 0.84 | 0.79 |
|  | Research method | 0.12 | 0.07 | 0.14 |
|  | Bitplane scrambling method | 0.56 | 0.38 | 0.48 |
| Diagonal axis | Original image | 0.86 | 0.95 | 0.8 |
|  | Research method | 0.07 | 0.15 | 0.12 |
|  | Bitplane scrambling method | 0.43 | 0.46 | 0.59 |
